# Evolution of a trait distributed over a large fragmented population: propagation of chaos meets adaptive dynamics

**DOI:** 10.1007/s00285-026-02371-9

**Published:** 2026-04-17

**Authors:** Amaury Lambert, Hélène Leman, Hélène Morlon, Josué Tchouanti

**Affiliations:** 1 Institut de Biologie de l’ENS (IBENS), École Normale Supérieure (ENS), CNRS UMR 8197, INSERM U1024, PSL University, Paris, France; 2 CASTING, Inria, Inserm, ENS de Lyon, Centre Léon Bérard, CNRS, UCBL1, Lyon, France; 3 Center for Interdisciplinary Research in Biology (CIRB), Collège de France, CNRS UMR 7241, INSERM U1050, PSL University, Paris, France; 4 ENS de Lyon, UMPA CNRS UMR 5669, Université de Lyon, Lyon, France; 5 Institut de Mathématiques de Toulouse, CNRS UMR 5219, Université de Toulouse, 31062 Toulouse Cedex 9, France

**Keywords:** Moran model, Metapopulation, Propagation of chaos, Adaptive dynamics, Trait substitution sequence, Canonical equation, Migration, Diffusion with jumps, Ecology, Macroevolution, 60F17, 60J25, 60J70, 60J76, 60K35, 35Q70, 92D15, 92D40

## Abstract

We consider a metapopulation made up of *K* demes, each containing *N* individuals bearing a heritable quantitative trait. Demes are connected by migration and undergo independent Moran processes with mutation and selection based on trait values. Mutation and migration rates are tuned so that each deme receives a migrant or a mutant in the same slow timescale and is thus essentially monomorphic at all times for the trait value (adaptive dynamics). In the timescale of mutation/migration, the metapopulation can then be seen as a giant spatial Moran model with size *K* that we characterize. As $K\rightarrow \infty$ and physical space becomes continuous, the empirical distribution of the trait value (over the physical and trait spaces) evolves deterministically according to an integro-differential evolution equation. In this limit, the trait value of every migrant is drawn from this global distribution, so that conditional on its initial state, trait values from finitely many demes evolve independently (propagation of chaos). Under mean-field dispersal, the value $X_t$ of the trait at time *t* and at any given location has a law denoted $\mu _t$ and a jump kernel with two terms: a mutation-fixation term and a migration-fixation term involving $\mu _{t-}$ (McKean–Vlasov equation). In the limit where mutations have small effects and migration is further slowed down accordingly, we obtain the convergence of *X*, in the new migration timescale, to the solution of a stochastic differential equation which can be referred to as a new, canonical jump-diffusion of adaptive dynamics. This equation includes an advection term representing selection, a diffusive term due to genetic drift, and a jump term, representing the effect of migration, to a state distributed according to its own law.

## Introduction

To understand the origin of the diversity, through time and among taxa, of a phenotypic trait (body mass, beak shape, leaf size, wing color...) is one of the central questions in evolutionary biology. Several approaches to address this question exist, depending on the scale studied.

On the slowest timescale, i.e., the scale of paleontology and macroevolution, the evolution of a trait, or rather of its species/population average, is frequently modeled by a stochastic process such as Brownian motion (Felsenstein 1973; Hansen and Martins 1996), the Ornstein–Uhlenbeck process (Hansen 1997; Butler and King 2004) or a Lévy process (Landis et al. 2013; Manceau et al. 2020). This top-down approach notably serves in comparative phylogenetic methods for the inference of macro-evolutionary dynamics from the knowledge of traits of present or fossil species, see (Pennell and Harmon 2013) for a review.

On the fastest timescale, i.e., the scale of a few generations, the distribution of a quantitative trait in a population is often assumed to be normal and its evolution is described through the dynamics of its mean and variance.

In quantitative genetics, the value of a trait is the sum of an environmental component and of a genetic, heritable component, often modeled as the sum of allelic effects at different loci. Selection on the trait value (assumed here to be one-dimensional) is typically modeled by a Gaussian-like fitness function of the trait value (stabilizing selection) with variance $\omega ^2$ and a possibly moving optimum (directional selection). Russell Lande and his coauthors showed in a series of foundational papers (Lande 1976, 1979; Lande and Arnold 1983; Bürger and Lande 1994) that under these normality assumptions, the average trait value follows an autonomous equation pushing it toward the optimum, with a Gaussian deviation with second central moment *A*/*N* due to genetic drift, where *A* is an increasing function of the additive genetic variance $\sigma _g^2$, with $A(0)=\omega ^2/2$ (in the absence of environmental noise) and $A(x)\sim x$ when *x* is large. The additive genetic variance $\sigma _g^2$ measures the degree of heritable polymorphism in the population. Its value at equilibrium decreases with population size/strength of selection (i.e., increases with $\omega$) and increases with mutation rate. It is essentially zero when selection is strong enough to deplete diversity and mutation rate is too small to replenish it.

In this latter case, one observes a separation of timescales: ecological dynamics occur quickly while mutations are rare, so that only a few trait values coexist at any given time. The theory of adaptive dynamics pushes this assumption to the extreme case where fixation occurs before the next mutation event and the average trait becomes the only trait value in the population, except during the short period when the resident trait value and the mutant trait value compete. Under this assumption, Metz et al. (1995) and Dieckmann and Law (1996) derived what is known as the canonical equation of adaptive dynamics, which describes the evolution of the so-called dominant trait value in the timescale of mutations. Later, Champagnat et al. (2006) and Baar et al. (2017) formally derived this equation in the context of large populations.

At the cost of suppressing all the information on polymorphism, this alternative approach offers several benefits: (1) by considering two-player games (resident vs. mutant), it allows for richer ecological dynamics than stabilizing selection resulting from an absolute fitness function, (2) the fitness of the mutant in the resident background emerges naturally from the ecology (see below) rather than being given a priori, and (3) the evolution of the dominant trait in the population can be rigorously derived mathematically, without the normality assumptions required for the average trait value in a polymorphic population to have autonomous dynamics. See the companion paper (Lambert et al. 2024) for a tentative synthesis.

Although adaptive dynamics theory traditionally deals with large populations, the assumption that fixation occurs rapidly compared to mutations should be more relevant for small populations. Champagnat and Lambert (2007) extended the approach of adaptive dynamics to finite populations, where the possible fixation of trait values with suboptimal fitness introduces stochasticity into the canonical equation of adaptive dynamics. Here, the fitness of the mutant is its probability of fixation in a resident population at stochastic equilibrium. In the limit of small mutational effects, the dynamics of the trait value are then described by a diffusion process, called the canonical diffusion of adaptive dynamics, with a similar advection term as derived by Dieckmann and Law (1996), as well as a diffusion term due to genetic drift. As in Lande (1976), this diffusion term scales like 1/*N*, but it depends on the covariance matrix of the mutation kernel rather than on the Hessian of the fitness function at the optimum.

The two approaches (quantitative genetics and the canonical diffusion of adaptive dynamics) have the merit to unveil the micro-evolutionary underpinnings of trait evolution but suffer from inherent contradictions regarding population size: The first one (quantitative genetics) needs population size to be both finite (for genetic drift to play a role) and infinite (for normality of trait distribution to hold, which is required for the dynamics of the average trait to be autonomous). The second one (the canonical diffusion of adaptive dynamics) needs population size to be small (for fixation of slightly deleterious mutations to be possible and fast) but large enough to avoid extinction. In addition, both approaches assume that the population is panmictic, although this may be a reasonable assumption only on the scale of a few *N* generations, as panmixia cannot last on macroevolutionary timescales, e.g., where speciation events are likely to occur.

Introducing spatial structure and environmental heterogeneity into the canonical diffusion of adaptive dynamics offers a potential solution to these issues. In this article, we consider a population made up of a large number *K* of demes or patches each harboring the same finite number *N* of individuals and connected by migration. We can thus let *K* be large and even tend to $\infty$ so as to get a continuous distribution of trait values, but leave *N* finite, so as to keep sources of stochasticity potentially playing a role at the macroevolutionary scale: genetic drift (in each deme) and local sampling.

We analyze different scalings of this spatially structured, microscopic model, progressively considering rare mutations, a large number of patches and finally small mutational effects, sticking to the adaptive dynamics framework.

In the first part of the paper, we focus on the rare mutation limit. By appropriately rescaling time, we derive a limit described by a series of connected Trait Substitution Sequences (TSS), similar to the results of Champagnat and Lambert (2007) and Lambert et al. (2024). Beyond this, we introduce a more innovative scaling, where the number *K* of patches becomes large while keeping the number *N* of individuals per patch finite. In this regime, we analyze the coupling of the total metapopulation dynamics with the dynamics of any finite subset of patches. This result is notable for two key reasons. First, it connects to the theory of propagation of chaos, yielding a novel limiting process characterized by jumps in its dynamics (Sznitman 1991; Chaintron and Diez 2022b, a). Second, it provides a justification for the structured metapopulation models introduced by Gyllenberg et al. (1997), which have been extensively used in spatial ecology. These models simplify metapopulation dynamics into two levels: deterministic macroscopic dynamics for the entire metapopulation and stochastic local dynamics within individual patches. For previous works on those topics, we refer the reader to (Del Moral and Miclo 2000; Hutzenthaler and Pieper 2020; Cloez and Corujo 2022; Cordero et al. 2026) for propagation of chaos in population genetic models and to (Holyoak and Leibold 2005; Lehmann 2008; Allen et al. 2013; Papaix et al. 2013; Wakano and Lehmann 2014) for adaptive dynamics in a metapopulation setting.

In the second part of the paper, we introduce the assumption of *small mutations* and examine two timescales. The term *small mutations* is taken from previous articles dealing with similar issues (see Champagnat et al. 2001, 2006; Champagnat and Lambert 2007) and refers to mutations with very weak phenotypic effects. Considering this additional assumption and that we do not rescale time, mutants with significantly different trait value do not have time to arise and we are left, under mean-field dispersal, with a multitype, antisymmetric Lotka-Volterra system. Although analyzing this system is nontrivial, as we discuss in the main text, we provide insights into specific cases.

If we rescale time so as to see new mutant trait values arise, we need to simultaneously rescale migration to get convergence to an equation that can be interpreted as a canonical equation of adaptive dynamics for metapopulations. As before, this limiting process operates on two levels: a macroscopic metapopulation level and a local patch level. At the local level, the dynamics are driven by a diffusion process with jumps–a feature, to the best of our knowledge, not previously observed.

The paper is organized as follows. In Sect. 2, we present the microscopic model and the main assumptions. Section 3 displays the results regarding the limiting process when assuming rare mutations and migrations. In Sect. 4, we present a result of propagation of chaos when the number of patches grows to infinity. In Sect. 5, we finally add an assumption of small mutations and give two possible limiting processes. If the migration rate is not small, there already exists an interesting behavior to study in the natural time scale, which is presented in Sect. 5.1. Otherwise, if the migration rate is small, we derive a canonical equation of adaptive dynamics with diffusion and jumps by rescaling time. This final result is presented in Sect. 5.2. Finally, Sect. 6 is devoted to the proofs.

## Modeling assumptions

We consider a metapopulation consisting of *K* interacting demes, also called patches or sites, labelled $\ell =1,\ldots ,K$, each containing a population of fixed size $N\ge 1$. The $\ell$-th patch is composed of individuals with labels $i=(\ell -1)N+1,\ldots ,\ell N$ and characterized by their phenotypic traits $x^i\in {\mathbb {R}}^d$. The joint dynamics of trait values follow a multivariate time-continuous birth–death process that can be seen as *K* coupled Moran models (Moran 1958):

- **Within-patch resampling.** For each pair of individuals with trait values *x* and *y* in the $\ell$-th patch, the individual with trait value *x* is replaced with a new individual with trait value *y*, at rate $c(\frac{\ell }{K},x,y)>0$, which may depend on the two trait values *x* and *y* (selection) and on the patch label $\ell$ (spatial heterogeneity);
- **Mutation.** Each individual with trait value *x* in the $\ell$-th patch mutates at rate $\gamma \theta (\frac{\ell }{K},x)\ge 0$, and acquires a new trait value chosen according to the law $Q(\frac{\ell }{K},x,dy)$, which may depend on the former trait value *x* and on the patch label $\ell$;
- **Migration.** For each pair of individuals with trait values *x* and *y* and belonging to patches $\ell$ and $\ell '$ respectively, the individual with trait value *x* in the $\ell$-th patch is replaced with an individual with trait value *y* at rate $\gamma \lambda ((\frac{\ell }{K},x),(\frac{\ell '}{K},y))/K\ge 0$.

Notice that our notation allows us to embed the patches into the interval [0, 1]. As long as *K* is finite, this has no impact on the migration structure, as the functions can be defined arbitrarily at the points $\{1/K,2/K,..,1\}$ and then be extended on [0, 1] with any needed smoothness. However, when *K* tends to $\infty$, this embedding suggests a rigid spatial structure. Namely, each deme is in the limit $K\rightarrow \infty$ reduced to a point in continuous space and each migration from *r* to $r'$ is ruled by a dispersal kernel $\lambda ((r,x), (r',y))$, as in graphons (Athreya et al. 2021), possibly depending on the traits *x* and *y* held at *r* and $r'$.

From now on, we make the following assumptions:

### Assumption 1

The maps *c*, $\theta$ and $\lambda$ are non-negative, bounded and measurable.

Notice that this model can incorporate spatially heterogeneous mechanisms of selection, mutation and migration by allowing all kernels to depend on patch labels and trait values. Notice also that we included a scaling parameter $\gamma$, which will be assumed to converge to 0 in order to study the limit of rare mutations and migrations. The scaling of the migration kernel by *K* puts mutations and migrations in the same time scale, even when *K* is large. In this framework, which resembles the assumptions of adaptive dynamics (Dieckmann and Law 1996), we expect low levels of diversity in each patch. If $\gamma$ is small enough, in each patch with finite population size *N*, fixation of one single trait value will even occur before the next event of mutation or migration, ensuring that in the mutation/migration timescale, all individuals of the same patch carry the same trait value, that we call *dominant*. Our goal is to describe the joint dynamics of dominant trait values over the metapopulation. In Sect. 3, we will keep the number *K* of patches finite, then in Sects. 4 and 5, we will let $K\rightarrow \infty$.

Let us describe the total population, present in the entire metapopulation, using the measure-valued stochastic process defined by

2.1 $$\begin{aligned} \nu ^{\gamma ,K}_t = \frac{1}{K}\sum _{\ell =1}^K\frac{1}{N}\sum _{i=1}^{N}\delta _{(\frac{\ell }{K},x^{H^\ell (i)}_t)} , \forall t\ge 0 \end{aligned}$$

where $H^\ell (i)=i + (\ell -1)N$ is the label of the *i*-th individual in the $\ell$-th patch, and $x^{H^\ell (i)}_t\in {\mathbb {R}}^d$ denotes its phenotypic trait value at time $t\ge 0$. This process describes the distribution of traits in the entire metapopulation. More specifically, it is a càd-làg Markov process with values in

2.2 $$\begin{aligned} {\mathcal {M}}^K_1 = \left\{ \frac{1}{NK}\sum _{i=1}^{NK} \delta _{(\frac{\ell ^i}{K},x^i)}, \text { with }x^i\in {\mathbb {R}}^d, \ell ^i\in \llbracket 1,K\rrbracket \right\} \subset {\mathcal {M}}_1({\mathcal {X}}) \end{aligned}$$

where ${\mathcal {X}}= [0,1]\times {\mathbb {R}}^d$, $\llbracket 1,K\rrbracket$ represents the set of integers $\{1,2,..,K\}$ and ${\mathcal {M}}_1({\mathcal {X}})$ is the set of probability measures on ${\mathcal {X}}$, endowed with the trace of the weak topology on the space ${\mathcal {M}}_F({\mathcal {X}})$ of finite measures on ${\mathcal {X}}$. We recall that, for any càd-làg process $(Y_t,t\ge 0)$ and for any $t> 0$, we will denote by $Y_{t-}$ the limit of $Y_s$ as *s* increases in value approaching *t*, i.e. $Y_{t-}:=\lim _{s\rightarrow t}Y_s$. For any topological space ${\mathcal {Y}}$, let us also define ${\mathcal {C}}_b({\mathcal {Y}})$ as the set of continuous and bounded real valued functions on ${\mathcal {Y}}$. According to our previous description of the process, we can define the process $(\nu ^{\gamma ,K}_t)_{t\ge 0}$ through its infinitesimal generator, that is, for any test function $\varphi \in {\mathcal {C}}_b({\mathcal {M}}^K_1)$,

2.3 $$\begin{aligned} \begin{aligned} {\mathcal {L}}^{\gamma ,K}\varphi (\nu )&= NK\iint _{{\mathcal {X}}}\nu (dr,dx)\left( NK\iint _{{\mathcal {X}}}1\!\!1_{r'=r}\nu (dr',dy) \right) c(r,x,y)\\&\quad \times \left[ -\varphi (\nu ) + \varphi \bigg ( \nu - \frac{\delta _{(r,x)}}{NK} + \frac{\delta _{(r,y)}}{NK} \bigg ) \right] \\&\quad + NK\gamma \iint _{{\mathcal {X}}}\theta (r,x)\nu (dr,dx)\int _{{\mathbb {R}}^d}Q(r,x,dy)\\&\quad \times \left[ -\varphi (\nu ) + \varphi \bigg ( \nu - \frac{\delta _{(r,x)}}{NK} + \frac{\delta _{(r,y)}}{NK} \bigg ) \right] \\&\quad + N^2K\gamma \iint _{{\mathcal {X}}}\nu (dr,dx)\iint _{{\mathcal {X}}}1\!\!1_{r'\ne r}\nu (dr',dy)\lambda ((r,x),(r',y))\\ &\quad \times \left[ -\varphi (\nu ) + \varphi \bigg ( \nu - \frac{\delta _{(r,x)}}{NK} + \frac{\delta _{(r,y)}}{NK} \bigg ) \right] . \end{aligned} \end{aligned}$$

Under Assumption 1, it is standard, using Poisson Point Measures as in Fournier and Méléard (2004), that the generator given by (2.3) defines a unique stochastic Markov process $(\nu _t^{\gamma ,K}, t\ge 0)$ on ${\mathcal {M}}_1({\mathcal {X}})$.

## Coupled trait substitution sequences

In this section, our aim is to let $\gamma \rightarrow 0$, i.e., study the dynamics of the process in the limit of rare mutations and rare migrations. As explained previously, in this regime ($\gamma \rightarrow 0$), the time is sufficiently large between two successive mutation/migration events that, in these intervals, the patches behave like independent Moran models and should reach their stationary regime which is a monomorphic state. Accelerating time, we expect the process to converge to a jump process that describes the dynamics of the dominant trait value within each patch, with jump rates that inherently couple interactions across different patches. In the case of a single patch, this framework reduces to the Trait Substitution Sequence (TSS), originally introduced in Metz et al. (1995), and later formalized rigorously by Champagnat (2006). In this work, we are interested in the more intricate scenario involving multiple patches.

Before stating our result, we need to introduce $\alpha (r,y,x)$. It denotes the invasion probability of a single individual with trait value *y* in the patch *r* filled with a monomorphic population of *x*-individuals, in the absence of any mutations or migrations associated with patch *r*. In other words, it is the invasion probability of a population with trait value *y* starting with the initial condition $\delta _{y} + (N-1)\delta _x$. Within this context, the number $N^x_t$ of individuals with trait value *x* in patch *r* follows a birth–death process with the following transition rates

3.1 $$\begin{aligned} n \xrightarrow []{\text { jumps to }} \left\{ \begin{aligned} n+1&\text { at rate } c(r,y,x)n(N-n) \\ n-1&\text { at rate }c(r,x,y)n(N-n)\, . \end{aligned}\right. \end{aligned}$$

In this setting, $\alpha (r,y,x)$ is the probability that $N^x_t$ hits the absorbing state 0 for some $t\ge 0$ starting from $N^x_0=N-1$. Computing this probability with classical tools for birth–death processes, we easily get that $\alpha (r,y,x)=0$ if $c(r,x,y)=0$, and otherwise

$$ \alpha (r,y,x) = \left( \sum _{k=0}^{N-1}\left( \frac{c(r,y,x)}{c(r,x,y)} \right) ^k \right) ^{-1}, $$

which, as expected, equals the neutral fixation probability $\frac{1}{N}$ when $c(r,y,x)=c(r,x,y)$. The main result of this section thus reads as follows.

### Proposition 3.1

Assume that all patches are initially monomorphic and that Assumption 1 holds. Then the sequence $\left\{ \left( \nu ^{\gamma ,K}_{t/\gamma }\right) _{t\ge 0}, \gamma >0\right\}$ converges in the sense of finite-dimensional distributions as $\gamma \rightarrow 0$ to the process $\nu ^K_t = \frac{1}{K}\sum _{\ell =1}^K\delta _{(\ell /K,X^{\ell ,K}_t)}$, where $X^K = (X^{1,K},\ldots ,X^{K,K})$ is a $({\mathbb {R}}^d)^K-$valued pure-jump Markov process described by the following transition rates

3.2 $$\begin{aligned}&x \xrightarrow []{\text { jumps to }}\left\{ \begin{array}{lll} \displaystyle \left( x^1,\ldots ,x^{\ell -1},y,x^{\ell +1},\ldots ,x^K \right) & \text {at rate }N\,\theta (\frac{\ell }{K},x^\ell ) \,\alpha (\frac{\ell }{K},y,x^\ell )& \, Q(\frac{\ell }{K},x^\ell ,dy), \\ \displaystyle \left( x^1,\ldots ,x^{\ell -1},x^{\ell '},x^{\ell +1},\ldots ,x^K \right) & \text {at rate } \frac{N^2}{K}\,\lambda ((\frac{\ell }{K},x^\ell ), (\frac{\ell '}{K},x^{\ell '}))\, & \alpha (\frac{\ell }{K},x^{\ell '},x^{\ell }), \end{array}\right. \end{aligned}$$

3.3 $$\begin{aligned}&\ell , \ell '=1,\ldots ,K, \text {independantly}. \end{aligned}$$

Observe how the fixation probability $\alpha (r, y,x)$ is integrated into the two jump rates, illustrating the long-term effect of selection on an individual bearing a new trait value appearing in a patch–whether through mutation or migration. Specifically, the jump rates reveal that the fate of the new trait depends on its ability to invade: if it successfully invades, it replaces the current dominant trait value, an event known as ‘fixation’; otherwise, it fails to establish and the mutation/migration event has no effect. These dynamics capture the selective pressures governing trait dominance within patches.

The proof of this result is an adaptation of the one developed by Champagnat (2006), Champagnat and Lambert, 2007, Theorem 3.1 and Lambert et al. (2024). We thus refer to these articles and do not detail the proof here.

Such a limiting model has been for example studied in Marrec et al. (2021) to understand how a population structure can impact the selection forces.

### Remark 3.2

In the timescale of mutations/migrations, the dynamics of the trait distribution across the metapopulation can be interpreted as a modified Moran model, where each individual corresponds to a site, and its trait value corresponds to the dominant trait value in this patch. This new Moran model features two kinds of events, substitution events occurring within each site, corresponding to events of mutation followed by fixation of the mutant, known in adaptive dynamics as “Trait Substitution Sequence”, and resampling events between sites, corresponding to events of migration followed by fixation of the migrant. Their jump kernels are the following:

- The substitution jump kernel in site $\ell$:
  3.4 $$\begin{aligned} N\,\theta (\frac{\ell }{K},x)\,\alpha (\frac{\ell }{K},y,x)\, Q(\frac{\ell }{K},x,dy) \end{aligned}$$
- The resampling jump kernel between sites $\ell$ and $\ell '$:
  3.5 $$\begin{aligned} \frac{N^2}{K}\,\lambda \left( (\frac{\ell }{K},x),(\frac{\ell '}{K},y)\right) \,\alpha (\frac{\ell }{K},y,x). \end{aligned}$$

## Large number of patches and propagation of chaos

We are now interested in studying the above limiting process, which corresponds to a collection of coupled Trait Substitution Sequences, under the assumption that the metapopulation is large, i.e., $K\rightarrow \infty$. The central questions we aim to address are how phenotypic traits evolve within a single patch, or in a finite set of patches, and how they collectively behave at the level of the metapopulation.

We start from the process $\nu ^K_t = \frac{1}{K}\sum _{\ell =1}^K\delta _{(\frac{\ell }{K},X^{\ell ,K}_t)}$ obtained in Proposition 3.1 and we fix, for any *K*, a finite number *J* of patches, with labels denoted $\ell ^K_1,\ldots ,\ell ^K_J\in \llbracket 1,K\rrbracket$. Before going further, let us make the following assumption.

### Assumption 2

 

- $(\nu ^K_0)_{K\ge 1}$ converges in law as $K\rightarrow +\infty$ towards a random variable $\nu _0$ with values in ${\mathcal {M}}_1({\mathcal {X}})$.
- For any $1\le j\le J$, there exist a (deterministic) $r^j\in [0,1]$ and an ${\mathbb {R}}^d$-r.v. $X^{j}_0$ such that
  $$\begin{aligned} \ell ^K_j/K\underset{K\rightarrow +\infty }{\longrightarrow }\ {r^j} \quad \text {and} \quad X^{\ell ^K_j,K}_0 \underset{K\rightarrow +\infty }{\longrightarrow }\ {X^{j}_0} \text { in law.} \end{aligned}$$
- The functional rates $\theta (\cdot )$, $\lambda (\cdot )$ and $\alpha (\cdot )$ are continuous w.r.t. all variables. The mutation kernel is such that $(r,x)\mapsto \int _{{\mathbb {R}}^d}f(r,y)Q(r,x,dy)$ is continuous w.r.t. both variables for any continuous and bounded function $f\in {\mathcal {C}}_b({\mathcal {X}})$.

By noticing that $\nu ^K_0(dr,{\mathbb {R}}^d) = \frac{1}{K}\sum _{\ell =1}^K \delta _{\ell /K}(dr)$, the first point of Assumption 2 above implies in particular that the limiting r.v. satisfies $\nu _0(dr,{\mathbb {R}}^d)=dr$ a.s.. This is true as we assume that all patches have the same size. This assumption could eventually be relaxed, assuming for example that $\nu _0(dr,{\mathbb {R}}^d)$ has a.s. a density with respect to Lebesgue measure. In this case, the results presented in this paper should still hold. However to avoid unnecessary complexity, we restrict our focus on the case of patches of the same size.

The first result below gives an answer to our main questions of the section in the general case. The following propositions will then refine and reinforce these results in the special cases of initial independence and spatial homogeneity. Let us start by stating the general result.

### Theorem 4.1

Under Assumptions 1 and 2, the sequence $\{ (X^{\ell ^K_1,K},\ldots , X^{\ell ^K_J,K},\nu ^K), K\ge 1 \}$ converges in law in the Skorohod space ${\mathbb {D}}([0,T],({\mathbb {R}}^d)^J\times {\mathcal {M}}_1({\mathcal {X}}))$. Its limit process $(X^1,\ldots ,X^J,\nu )$ has the initial condition $(X^1_0,\ldots ,X^J_0,\nu _0)$. $\nu$ is the unique ${\mathcal {C}}([0,T],{\mathcal {M}}_1({\mathcal {X}}))-$valued solution to the following weak equation defined, for any test function $\varphi \in {\mathcal {C}}_b({\mathcal {X}})$, by

4.1 $$\begin{aligned} \begin{aligned} \frac{d}{dt}\iint _{{\mathcal {X}}}\varphi (r,x)\nu _t(dr,dx)&= N\iint _{{\mathcal {X}}} \theta (r,x)\nu _t(dr,dx)\\ &\quad \quad \int _{{\mathbb {R}}^d}Q(r,x,dy)\alpha (r,y,x)\big [ \varphi (r,y) - \varphi (r,x) \big ] \\&\quad \quad + N^2\iint _{{\mathcal {X}}}\nu _t(dr,dx)\\ &\quad \quad \iint _{{\mathcal {X}}}\nu _t(dr',dy)\lambda \big ((r,x),(r',y)\big )\alpha (r,y,x)\big [ \varphi (r,y) - \varphi (r,x) \big ] \end{aligned} \end{aligned}$$

and $(X^1,\ldots ,X^J)$ is an $({\mathbb {R}}^d)^J-$valued time-inhomogeneous pure-jump Markov process, where at any time *t*, independently for any $j\in \{1,..,J\}$, $(x^1,\ldots , x^J)$ jumps to $\left( x^1,\ldots ,x^{j-1},y,x^{j+1},\ldots ,x^J \right)$ at rate

4.2 $$\begin{aligned} N\,\theta (r^j,x^j)\,\alpha (r^j,y,x^j)\, Q(r^j,x^j,dy) +N^2\int _0^1\lambda ((r^j,x^j),(r',y))\,\alpha (r^j,y,x^j)\, {\nu _{t}}(dr',dy). \end{aligned}$$

No other jump occurs.

### Proof

The proof relies on standard techniques developed by Ethier and Kurtz (1986) and (Bansaye and Méléard (2015)) (see the proof of Theorem 7.4 for example). It goes through two steps: tightness and identification of the limit. We give an overview of the two steps here and complementary arguments can be adapted from the references given above.

As a first step, the tightness in ${\mathbb {D}}([0,T],({\mathbb {R}}^d)^J\times {\mathcal {M}}_1({\mathcal {X}}))$ of the sequence of trajectory laws is proved. To this aim, the papers of Roelly-Coppoletta (1986) and Méléard and Roelly (1993) ensure that we can focus on the study of the tightness of $\{ (X^{\ell ^K_1,K},\ldots , X^{\ell ^K_J,K},\langle \nu ^K,\varphi \rangle ), K\ge 1 \}$ for all $\varphi \in {\mathcal {C}}_b({\mathcal {X}})$. Then, the tightness of the semi-martingales is obtained using the criterion defined by Aldous (1978) and Rebolledo (Joffe and Métivier 1986) (see the proof of Theorem 7.4 in Bansaye and Méléard (2015) for more details). Tightness thus ensures the existence of a subsequential limit by Prokhorov’s Theorem. Noticing that ${\mathbb {E}}\left[ \sup _{0\le t\le T}|\langle \nu ^K_{t} - \nu ^K_{t-},\varphi \rangle | \right] \le \frac{2\Vert \varphi \Vert _\infty }{NK}\rightarrow 0$ as $K\rightarrow +\infty$ for any $\varphi \in {\mathcal {C}}_b({\mathcal {X}})$, we deduce that each subsequential limit is continuous and then belongs to ${\mathcal {C}}([0,T],{\mathcal {M}}_1({\mathcal {X}}))$.

In the second step, the limit is identified by proving first that any subsequential limit satisfies the equation (4.1) and the description given in (4.2). Then the uniqueness of the solution of (4.1) is shown, which ensures that all limit distributions are identical and entails the convergence. $\square$

The limiting process aligns closely with the structured metapopulation models introduced by Gyllenberg et al. (1997) and widely applied in spatial ecology, including works by Gyllenberg and Metz (2001) and more recently Brian et al. (2023). These models operate on two levels: a local level, which may be stochastic and captures the dynamics within a single patch, allowing for finite local population sizes; and a metapopulation level, which is defined as the distribution of the local states, implicitly assuming an infinite number of patches. The limiting process described here fits entirely within this framework, and the result is thus establishing a connection between microscopic models and macroscopic structured metapopulation models.

Note that the measure-valued limit process $(\nu _t)_{t\ge 0}$ is fully characterized by Eq. (4.1) which is closed and that its dynamics is deterministic, so that the only randomness in $\nu _t$ comes from its starting point $\nu _0$. Also notice that the trait values $(X^1,\ldots ,X^J)$ hosted at sites $(r^1,\ldots ,r^J)$ are independent conditional on the process $(\nu _t)_{t\ge 0}$ and the independence of the initial values. As a consequence, if $\nu _0$ is not random, then $\nu _t$ is not random and the trajectories $(X^1,\ldots ,X^J)$ are independent as soon as their initial values are, which is the case when $X^{1}_0,\ldots ,X^J_0$ have deterministic values for example. We record this fact in the following statement.

### Corollary 4.2

(Asymptotic independence) Assume that $X^{1}_0,\ldots ,X^J_0$ are independent and $\nu _0$ is deterministic, then under the assumptions of Theorem 4.1, the limit process $(X^1,\ldots ,X^J,\nu )$ is such that $X^1,\ldots ,X^J$ are independent and $\nu$ is deterministic.

We will now see that in the case of a homogeneous structure, the result can be simplified and refined. Let us first state what we mean by homogeneity.

### Definition 4.3

The metapopulation is said to be homogeneous iff the functional parameters that govern local dynamics and migration do not depend on patch labels.

Under this condition, patches are exchangeable in the metapopulation and for the sake of simplicity we adopt a notation of the functional parameters where we merely drop the dependency upon the patch label. In this context, we have the following corollary.

### Corollary 4.4

(Propagation of chaos) Assume that the metapopulation is homogeneous and that $X^{1,K}_0,\ldots ,X^{K,K}_0$ are i.i.d with common distribution $\mu _0\in {\mathcal {M}}_1({\mathbb {R}}^d)$. Then under the assumptions of Theorem 4.1, the processes $X^1,\ldots ,X^J$ are i.i.d copies of an ${\mathbb {R}}^d-$valued pure-jump time-inhomogeneous Markov process *X* with law at time *t* denoted $\mu _t$ and jump kernel at time *t*

4.3 $$\begin{aligned} N\theta (x)\alpha (y,x)Q(x,dy) + N^2\lambda (x,y)\alpha (y,x)\mu _{t}-(dy) \end{aligned}$$

In addition, $\mu _t$ is continuous in time and the global trait value distribution is given by $\nu _t(dr,dx)=dr\mu _t(dx)$ on ${\mathcal {X}}$.

The notion of propagation of chaos describes a phenomenon where, as the total number of particles in a system becomes large, any finite subset of particles become asymptotically independent, and their joint distribution converges to the product of identical marginal distributions governed by a limiting law. The stochastic process *X* introduced in this result is known as a McKean–Vlasov process (Sznitman 1991): its jumps at any given time $t>0$ depend on the distribution of $X_{t-}$. In particular, it is easy to verify that if $\mu _0(dx) = g(0,x)dx$ and $Q(x,dy) = q(x,y)dy$, then $\mu _t(dx)$ admits a density *g*(*t*, *x*) with respect to the Lebesgue measure. As a consequence, we obtain the following representation of the trajectories of *X*

4.4 $$\begin{aligned} \begin{aligned} dX_t&= \int _{{\mathbb {R}}_+\times {\mathbb {R}}_+\times {\mathbb {R}}^d} (y-X_{t-})1\!\!1_{z\le N\theta (X_{t-})\alpha (y,X_{t-})q(X_{t-},y)} \pi _1(dz,dy,dt)\\&~ + \int _{{\mathbb {R}}_+\times {\mathbb {R}}_+\times {\mathbb {R}}^d} (y-X_{t-})1\!\!1_{z\le N^2\lambda (X_{t-},y)\alpha (y,X_{t-})g(t,y)} \pi _2(dz,dy,dt) \end{aligned} \end{aligned}$$

where $\pi _1(dz,dy,dt), \pi _2(dz,dy,dt)$ are two independent Poisson point measures with common intensity the Lebesgue measure on ${\mathbb {R}}_+\times {\mathbb {R}}^d\times {\mathbb {R}}_+$.

## Small mutations

We are now interested in studying the process defined by (4.1) and (4.2) in the limit of small mutation steps. To this aim, we consider in all that follows that there exists a centered probability kernel *m*(*r*, *x*, *dh*) such that

5.1 $$\begin{aligned} Q^\varepsilon (r,x,dy) := (\tau _xm)(r,x,\frac{dy}{\varepsilon }), \forall r\in [0,1],\forall x\in {\mathbb {R}}^d, \end{aligned}$$

where $\tau _x m$ is the shift map by *x*. In other words, the law *m*(*r*, *x*, *dh*) is the probability distribution of the scaled mutation step, so that $y\sim Q^\varepsilon (r,x,dy)$ is equivalent to $y=x + \varepsilon h$ where $h\sim m(r,x,dh)$.

Section 5.1 presents the convergence of the process (4.1) as $\varepsilon$ goes to 0. It shows that, because significantly different trait values take too long to arise by mutation, the overall diversity thus can only remain constant or decrease over time. The dynamics of the frequencies of the initially present trait values are then analyzed, with further refinement in the case of a homogeneous population.

Section 5.2 explores what happens when time is accelerated enough to see new mutant traits emerge. To obtain convergence to a proper limiting process, one needs to rescale migration rates to slow migration before accelerating time. This framework is fully in alignment with the study of the canonical equation of adaptive dynamics, as explained below.

### No time acceleration

Let us start with the study of the case where mutation effects vanish but time is not accelerated so as to see the emergence of significantly different mutant trait values. This study is based on the simple hypothesis that the scaled mutation step has a bounded moment, that is:

#### Assumption 3

For any $\varepsilon >0$, the mutation kernel $Q^\varepsilon$ is defined by Equation (5.1) and there exists $\beta \in (0,1]$ such that

5.2 $$\begin{aligned} \sup _{(r,x)\in [0,1]\times {\mathbb {R}}^d}\int _{{\mathbb {R}}^d}\Vert h\Vert ^\beta m(r,x,dh) < +\infty . \end{aligned}$$

We also add some classical assumptions about the parameters:

#### Assumption 4

We assume that

- $\theta \in {\mathcal {C}}_b({\mathcal {X}})$, with ${\mathcal {C}}_b({\mathcal {X}})$ the set of continuous and bounded functions on ${\mathcal {X}}$,
- The map $((r,x),(r',y))\rightarrow \lambda (r,x,r',y)\alpha (r,y,x)$ belongs to ${\mathcal {C}}^{\beta }_b({\mathcal {X}}\times {\mathcal {X}})$, the set of $\beta$-Hölder and bounded functions on ${\mathcal {X}}\times {\mathcal {X}}$. To simplify notation, we will sometimes write $\lambda \alpha \in {\mathcal {C}}^\beta _b({\mathcal {X}}\times {\mathcal {X}})$ to refer to this assumption.

Under Assumption 4, we easily verify that the sequence of processes $(\nu ^\varepsilon )_{\varepsilon >0}$, solutions of Eq. (4.1), converges in ${\mathcal {C}}([0,T],{\mathcal {M}}_1({\mathcal {X}}))$ as $\varepsilon \rightarrow 0$ towards a limit from which mutations are absent. To be more specific, we introduce the usual norm on the Hölder-Zygmund space ${\mathcal {C}}^{\beta }_b({\mathcal {X}})$ defined by

$$\begin{aligned} \Vert \varphi \Vert _{{\mathcal {C}}^\beta _b({\mathcal {X}})} = \Vert \varphi \Vert _\infty + \sup _{\begin{array}{c} (r,x),(r',y)\in {\mathcal {X}}\\ (r,x)\ne (r',y) \end{array}}\frac{|\varphi (r,x) - \varphi (r',y)|}{\left( |r-r'|+\Vert x-y\Vert \right) ^\beta } \end{aligned}$$

which allows us to define the following distance between two processes with values in the space of measures ${\mathcal {M}}_F({\mathcal {X}})$:

5.3 $$\begin{aligned} d_{\beta }(\mu ,\nu ) := \sup _{0\le t\le T}\sup _{\begin{array}{c} \varphi \in {\mathcal {C}}^{\beta }_b({\mathcal {X}}) \\ \Vert \varphi \Vert _{{\mathcal {C}}^{\beta }_b({\mathcal {X}})}\le 1 \end{array}}\left\{ \langle \mu _t,\varphi \rangle - \langle \nu _t,\varphi \rangle \right\} . \end{aligned}$$

The following statement is proved in Sect. 6.2.

#### Proposition 5.1

Assume that Assumptions 3 and 4 hold. For all $\varepsilon >0$, we define $(\nu ^\varepsilon _t)_{t\ge 0}$ as the solution to Eq. (4.1), with initial condition $\nu _0$. Then the sequence $(\nu _t^\varepsilon ,t\in [0,T])_{\varepsilon >0}$ converges when $\varepsilon$ tends to 0 toward $\nu$, the solution to the following equation, for any $\varphi \in {\mathcal {C}}({\mathcal {X}})$,

5.4 $$\begin{aligned} &  \frac{d}{dt}\iint _{{\mathcal {X}}}\varphi (r,x)\nu _t(dr,dx) = N^2\iint _{\mathcal {X}}\nu _t(dr,dx)\nonumber \\ &  \iint _{\mathcal {X}}\nu _t(dr',dy)\lambda ((r,x),(r',y))\alpha (r,y,x)\big [ \varphi (r,y) - \varphi (r,x) \big ], \end{aligned}$$

with initial condition $\nu _0$. The convergence holds in the sense that there exists $C_{\beta ,T}>0$ which satisfies for all $\varepsilon >0$,

5.5 $$\begin{aligned} d_{\beta }(\nu ^\varepsilon ,\nu ) \le C_{\beta ,T}\varepsilon ^\beta . \end{aligned}$$

Notice that the limiting dynamics are driven by events of migration and fixation, without any event of mutation. Hence, the diversity in the metapopulation can only remain constant or decrease. In particular, a metapopulation that is monomorphic from the start, i.e., $\nu _0(dr,dx) = \delta _{x^*}(dx)dr$ (with fixed $x^*\in {\mathbb {R}}^d$), remains constant, i.e., $\nu _t=\nu _0$ for all $t\ge 0$.

More interestingly, we can describe the dynamics of diversity when there are initially $n>1$ trait values present in the metapopulation. The following statement also is proved in Sect. 6.2.

#### Proposition 5.2

Assume an initial condition of the form $\nu _0(dr,dx) = \sum _{i=1}^nw^i_0(r)\delta _{x^i}(dx)dr$ where the $w^i_0(r)\ge 0$ and $\sum _{i=1}^n w^i_0(r)dr = dr$. The solution to (5.4) is then given by

$$\begin{aligned} \nu _t(dr,dx) = \sum _{i=1}^nw^i_t(r)\delta _{x^i}(dx)dr \end{aligned}$$

where for all $i=1,\ldots ,n$ and $r\in [0,1]$, the functions $w^i_.(r)$ are non-negative, such that $\forall t\ge 0, \sum _{i=1}^n w^i_t(r)dr = dr$ and are weak solutions to the system of integro-differential equations

5.6 $$\begin{aligned} \begin{aligned} \partial _tw^i_t(r)&= -N^2w^i_t(r)\sum _{j=1}^n\alpha (r,x^j,x^i)\int _0^1\lambda ((r,x^i),(r',x^j))w^j_t(r')dr' \\&\quad \quad + N^2\sum _{j=1}^nw^j_t(r)\alpha (r,x^i,x^j)\left( \int _0^1\lambda ((r,x^j),(r',x^i))w^i_t(r')dr' \right) . \end{aligned} \end{aligned}$$

Following the proof of this proposition, the result can be generalized under the weaker assumption that $\int _{0}^1\sum _{i=1}^n w^i_0(r)dr = 1$, yielding the same conclusion for any $t\ge 0$. This extension is particularly relevant when the assumption of uniform population sizes across patches is relaxed (see the comment below Assumption 2).

The dynamics given by (5.6) simplify even further when the metapopulation is homogeneous in the sense of Definition 4.3.

#### Corollary 5.3

Assume as in Proposition 5.2 that $\nu _0(dr,dx) = \sum _{i=1}^nw^i_0(r)\delta _{x^i}(dx)dr$ and define the *mean weight* of trait value $x^i$ at time *t* as

$$ \overline{w}^i_t := \int _0^1w^i_t(r)dr. $$

If the metapopulation is homogeneous, then the mean weights satisfy the following conservative replicator dynamics

5.7 $$\begin{aligned} \frac{d\overline{w}^i_t}{dt} = \overline{w}^i_t\sum _{j=1}^na_{ij}\overline{w}^j_t \qquad t\ge 0, i=1,\ldots ,n, \end{aligned}$$

where $a_{ij}=G(x^i,x^j)$ with $G(y,x) = N^2\big (\lambda (x,y)\alpha (y,x) - \lambda (y,x)\alpha (x,y)\big )$ for any $x,y\in {\mathbb {R}}^d$.

Note that the matrix $A=(a_{ij})_{\{1\le i\le n,1\le j\le n\}}$ is antisymmetric, because $G(y,x) = -G(x,y)$. The replicator dynamics (5.7) are also known as antisymmetric Lotka–Volterra equations and are the subject of a rich literature, see e.g. Bomze (1983), Durney et al. (2011), Knebel et al. (2013) and Geiger et al. (2018).

In general, it is difficult to precisely determine the long-term behavior of this system (5.7), and in particular to determine if all trait values coexist in steady state, as this is highly sensitive to the specific form of the interaction matrix *A*. To illustrate this dependency, we present particular cases, each leading to fundamentally different long-term dynamics. These examples highlight the critical role of *A* in shaping the evolution of the system (Fig. 2).

**Case 1. Competitive exclusion.** In this first case, we exhibit a general sufficient condition that implies the convergence of the system to a monomorphic state. More precisely, we can state the following corollary, which is proved in Sect. 6.2.

#### Corollary 5.4

Under the assumptions of Corollary 5.3, if there exists an integer $1\le i^\star \le n$ such that $\overline{w}^{i^\star }_0>0$ and

5.8 $$\begin{aligned} a_{i^\star j}>0\qquad j\ne i^\star , \end{aligned}$$

then the trait value $x^{i^\star }$ invades the metapopulation in the sense that $\overline{w}^{i^\star }_t\rightarrow 1$ and for all $j\ne i^\star$, $\overline{w}^{j}_t\rightarrow 0$ as $t\rightarrow \infty$.

The following example illustrates this corollary.

#### Example

Consider an example with trait values in [0, 1] such that $a_{ij}=x^j - x^i$. This occurs for example if $\lambda (x,y)\alpha (y,x) = x(1+y)/N^2$, so that

$$ G(y,x) = x(1+y) - y(1+x) = x - y, \qquad x,y\in [0,1]. $$

**Fig. 1:**
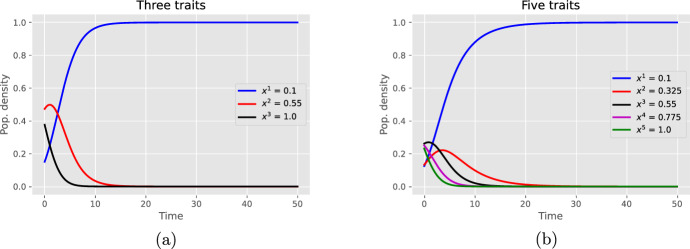
Invasion of the smallest trait value: **a** three distinct initial trait values; **b** five distinct initial trait values

As a consequence and according to Corollary 5.4, the smallest trait value will invade the metapopulation. Figure 1 shows typical simulations describing the dynamical system (5.7) in dimension $n=3,5$.

**Case 2. Cycling dynamics.** When there is no $i^\star$ that satisfies Eq. (5.8) of the previous corollary, then the model is more difficult to study and may often display cycling dynamics, as in the following example.

#### Example

In this example, we consider trait values in [0, 1] again and choose a migration-fixation rate given by $\lambda (x,y)\alpha (y,x) = \big (1+\sin (2\pi (x-y))\big )/N^2$. Then the fitness function satisfies

$$\begin{aligned} G(y,x) = 2\sin (2\pi (x-y)), \qquad x,y\in [0,1]. \end{aligned}$$

Note that the periodic behavior of solutions is not due to the periodic nature of *G*, which is only evaluated at the possible pairs of $n=$3 or 5 distinct trait values.

**Fig. 2:**
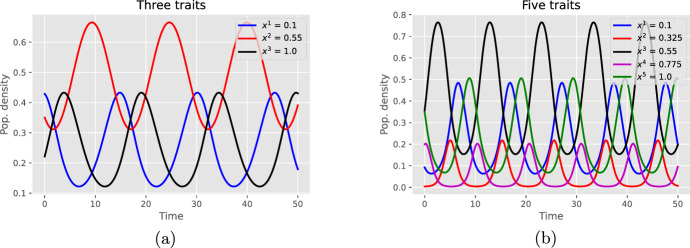
Cyclic trajectories: **a** three distinct initial trait values; **b** five distinct initial trait values

### Accelerating time, slowing down migration and the canonical diffusion with jumps

In this section, we assume as before that the mutation steps are given by (5.1), we additionally assume that the rate of migration is $\varepsilon ^2\lambda (\cdot )$ instead of $\lambda (\cdot )$ and we accelerate time in order to see the effect of mutations and of migrations, whose rates are tuned so as to become significant on the same timescale. As in the previous section, we study the limit as $\varepsilon$ goes to 0 of the process defined by (4.1) and (4.2). This approach allows us to derive a canonical equation, as mentioned in the introduction, that captures the dynamics of the dominant trait value within a single patch.

To this aim, let us first introduce some notation and functions that play an important role in the dynamics.

- The covariance matrix of the rescaled mutation steps for an individual with trait value $x\in {\mathbb {R}}^d$ located at position $r\in [0,1]$ is
  $$ \Sigma (r,x) := \left( \int _{{\mathbb {R}}^d}h_ih_jm(r,x,dh) \right) _{1\le i,j\le d}, $$
  which is a non-negative symmetric matrix. For any $(r,x)\in [0,1]\times {\mathbb {R}}^d$, we denote by $\sigma (r,x)$ the non-negative $d-$dimensional matrix such that $\Sigma (r,x) = \sigma (r,x)(\sigma (r,x))^T$.
- We denote by $\textrm{Fit}(r,y,x) = \log \left( \frac{c(r,x,y)}{c(r,y,x)}\right)$ the relative fitness of trait value $y\in {\mathbb {R}}^d$ compared to $x\in {\mathbb {R}}^d$ in a population located at position $r\in [0,1]$.
- Let $k\ge 2$ be an integer and let *f* be a differentiable real function of *k* variables. If the *i*-th variable of *f* is a *d*-dimensional vector, then we denote by $\nabla _i f$ the *d*-dimensional gradient of *f* with respect to its *i*-th component.
- Finally, we will denote by $(B_t)_{t\ge 0}$ a $d-$dimensional Brownian motion.

We make the following assumptions:

#### Assumption 5

For any $\varepsilon >0$, the mutation kernel $Q^\varepsilon$ is defined by Equation (5.1) and there exists $\beta \in (0,1]$ such that

5.9 $$\begin{aligned} \sup _{(r,x)\in [0,1]\times {\mathbb {R}}^d}\int _{{\mathbb {R}}^d}\Vert h\Vert ^{2+\beta }m(r,x,dh) < +\infty . \end{aligned}$$

We also assume the following technical hypotheses on the parameters of the model

#### Assumption 6

 

- The functional rates $\lambda (\cdot ),\theta (\cdot ), c(\cdot )$ are continuous and bounded.
- The functional rate $c(\cdot )$ is differentiable according to variables *x*, *y* so that $\nabla _2\alpha$ is bounded and $\beta -$Hölder continuous uniformly in $r\in [0,1]$, where $\beta$ is the same as in Assumption 5.
- Assume that the parameters functions are chosen such that $\theta (\cdot )\Sigma (\cdot )\cdot \nabla _2\textrm{Fit}(\cdot )$ and $\sqrt{\theta (\cdot )}\sigma (\cdot )$ are Lipschitz continuous according to variables *x*, *y* uniformly in $r\in [0,1]$.

We are able to prove the following result which describes the convergence of the time-scaled process defined by (4.1) and (4.2) for $J=1$. In other words, it describes the dynamics of the trait value present at any given location. More specifically, let us fix $z\in [0,1]$, then for any $\varepsilon >0$, we define $(X^\varepsilon ,\nu ^\varepsilon )$ such that $\nu ^\varepsilon$ is the unique solution to the weak equation defined, for any test function $\varphi \in {\mathcal {C}}_b({\mathcal {X}})$, by

5.10 $$\begin{aligned} \begin{aligned} \frac{d}{dt}\iint _{{\mathcal {X}}}\varphi (r,x)\nu ^\varepsilon _t(dr,dx)&= N\iint _{{\mathcal {X}}} \theta (r,x)\nu ^\varepsilon _t(dr,dx)\\ &\quad \times \int _{{\mathbb {R}}^d}\alpha (r,y,x)\big [ \varphi (r,y) - \varphi (r,x) \big ]Q^\varepsilon (r,x,dy) \\&\quad + N^2\!\iint _{{\mathcal {X}}}\!\nu ^\varepsilon _t(dr,dx)\!\iint _{{\mathcal {X}}}\!\varepsilon ^2\lambda \big ((r,x),(r',y)\big )\alpha (r,y,x)\\ &\quad \times \big [ \varphi (r,y) - \varphi (r,x) \big ]\nu ^\varepsilon _t(dr',dy) \end{aligned} \end{aligned}$$

and $(X^\varepsilon )$ is the time-inhomogeneous pure-jump Markov processes that jumps from *x* to *y* at infinitesimal rate

5.11 $$\begin{aligned} N\theta (z,x)\alpha (z,y,x) Q^\varepsilon (z,x,dy) + N^2\varepsilon ^2\int _0^1\lambda ((z,x),(r',y))\alpha (r,y,x) \nu ^\varepsilon _t(dr',dy). \end{aligned}$$

The next statement is proved in Sect. 6.3.

#### Theorem 5.5

Let $(X^\varepsilon _t,\nu ^\varepsilon _t)_{t\ge 0}$ be the process defined by (5.10) and (5.11) such that the sequence $\left\{ (X^\varepsilon _0,\nu ^\varepsilon _0), \varepsilon >0 \right\}$ converges in law towards $(X_0,\xi _0)$. Then under Assumptions 5–6, the sequence of processes $\{(X^\varepsilon _{\cdot /\varepsilon ^2},\nu ^\varepsilon _{\cdot /\varepsilon ^2}), \varepsilon >0\}$ converges in law as $\varepsilon \rightarrow 0$ in the Skorohod space ${\mathbb {D}}([0,T],{\mathbb {R}}^d\times {\mathcal {M}}_1({\mathcal {X}}))$. The limit process $(X,\xi )$ is a jump-diffusion process with initial condition $(X_0,\xi _0)$. It is the unique ${\mathbb {D}}([0,T],{\mathbb {R}}^d)\times {\mathcal {C}}([0,T],{\mathcal {M}}_1({\mathbb {R}}^d))-$valued solution of

5.12 $$\begin{aligned} dX_t = \frac{N-1}{2}\theta (z,X_t)\Sigma (z,X_t)\cdot \nabla _2\textrm{Fit}(z,X_t,X_t)dt + \sqrt{\theta (z,X_t)}\sigma (z,X_t)\cdot dB_t \end{aligned}$$

(where *B* is a $d-$dimensional Brownian motion), with jump kernel

5.13 $$\begin{aligned} N^2\int _0^1\lambda ((z,x),(r,y))\alpha (z,y,x)\xi _{t}(dr,dy), \end{aligned}$$

where for any test function $\varphi \in {\mathcal {C}}^2_b({\mathcal {X}})$,

5.14 $$\begin{aligned} &  \frac{d}{dt}\iint _{{\mathcal {X}}}\varphi (r,x)\xi _t(dr,dx) = N^2\iint _{{\mathcal {X}}}\xi _t(dr,dx)\nonumber \\  &  \quad \quad \times \iint _{{\mathcal {X}}}\xi _t(dr',dy)\lambda ((r,x),(r',y))\alpha (r,y,x)\big [ \varphi (r,y) - \varphi (r,x) \big ]\nonumber \\ &  \quad \quad +\iint _{{\mathcal {X}}} \left\{ \frac{N-1}{2}\theta (r,x)\left( \Sigma (r,x)\cdot \nabla _2\textrm{Fit}(r,x,x) \right) \cdot \nabla _x\varphi (r,x)\right. \nonumber \\  &  \quad \quad \left. + \frac{\theta (r,x)}{2}\sum _{ij}\Sigma _{ij}(r,x)\partial ^2_{x_ix_j}\varphi (r,x) \right\} \xi _t(dr,dx). \end{aligned}$$

Notice that the limit we identified corresponds to what is commonly referred to as a canonical equation (see, for example, Dieckmann and Law 1996; Champagnat et al. 2006; Champagnat and Lambert 2007). In our case, this limit is novel for at least two reasons: (1) it is stochastic and includes a diffusion term, which, to the best of our knowledge, has only been observed in Champagnat and Lambert (2007) and Lambert et al. (2024); and (2) it incorporates jumps arising from migration-fixation events.

#### Corollary 5.6

Assume that the metapopulation is homogeneous and that the initial condition $(X_0,\xi _0)$ is such that $\xi _0(dr,dx) = \mu _0(dx)dr$ where $\mu _0\in {\mathcal {M}}_1({\mathbb {R}}^d)$ is the law of $X_0$. Then under the assumptions of Theorem 5.5, the process *X* is a diffusion process with time-inhomogeneous jumps, with law at time *t* denoted $\mu _t$, solution to

5.15 $$\begin{aligned} dX_t = \frac{N-1}{2}\theta (X_t)\Sigma (X_t)\cdot \nabla _1\textrm{Fit}(X_t,X_t)dt + \sqrt{\theta (X_t)}\sigma (X_t)\cdot dB_t \end{aligned}$$

with jump kernel at time *t*

$$\begin{aligned} N^2\lambda (x,y)\alpha (y,x){\mu _{t}}(dy). \end{aligned}$$

We do not detail the proof of this corollary, which is similar to the one of Corollary 4.4 given in Sect. 6.1.

## Proofs

This section is devoted to the proofs of the paper. Since most of these proofs are adaptations of relatively classical results, we will primarily provide proof sketches, highlighting the key ingredients that support them.

### Proofs of section 4

As explained in the main text, we will not present the proof of Theorem 4.1 which is based on a classical argument of tightness and uniqueness of the limit. We will however describe sketches of proofs for the two following corollaries.

**Proof of Corollary** 4.2 The proof is relatively simple. Indeed, Theorem 4.1 ensures conditional independence of $X^1,..,X^J$ given *v*. Further, as soon as $\nu _0$ is deterministic, it follows immediately from (4.1) that the entire process $(\nu _t)_{t\ge 0}$ is deterministic. As a consequence, $X^1,\ldots ,X^J$ are independent inhomogeneous Markov processes.

**Proof of Corollary** 4.4 Let us assume now that the metapopulation is homogeneous in the sense of Definition 4.3 and that the initial conditions $X^{1,K}_0,\ldots ,X^{K,K}_0$ are i.i.d with common distribution $\mu _0\in {\mathcal {M}}_1({\mathbb {R}}^d)$.

On one hand, this assumption ensures that the limit process defined by (4.1) has the initial condition $\nu _0(dr,dx) = \mu _0(dx)dr$. Let us then define the measure valued deterministic process $(\mu _t(dx))_{t\ge 0}$ such that for all $t\ge 0$, $\mu _t(dx):=\int _0^1\nu _t(dr,dx)$. Then the uniqueness of the solution of (4.1) and the fact that the functions of the form $\varphi (x)\psi (r)$ are dense in ${\mathcal {C}}_b({\mathcal {X}})$ imply that $\nu _t(dr,dx) = \mu _t(dx)dr$ for any $t>0$.

On the other hand, notice that the initial conditions of $X^1,\ldots ,X^J$ are also i.i.d with common distribution $\mu _0$. In addition, these processes are independent (Corollary 4.2) with the same infinitesimal generator that is the one of the pure jump stochastic process *X* which jumps from *x* to *y* at the infinitesimal rate:

$$ N\theta (x)\alpha (y,x)Q(x,dy) + N^2\lambda (x,y)\alpha (y,x){\mu _{t}}(dy). $$

Let us now introduce the measure valued deterministic process defined by $\Lambda _t(dx) = {\mathbb {P}}(X_t\in dx|X_0\sim \mu _0)$. According to the Kolomogorov equations, it is the unique continuous process with initial condition $\mu _0$ satisfying for any $f\in {\mathcal {C}}_b({\mathbb {R}}^d)$

6.1 $$\begin{aligned} \begin{aligned} \frac{d}{dt}\int _{{\mathbb {R}}^d}f(x)\Lambda _t(dx)&= N\int _{{\mathbb {R}}^d}\theta (x)\Lambda _t(dx)\int _{{\mathbb {R}}^d}\alpha (y,x)Q(x,dy)[f(y)-f(x)] \\&\quad + N^2\int _{{\mathbb {R}}^d}\Lambda _t(dx)\int _{{\mathbb {R}}^d}\lambda (x,y)\alpha (y,x){\mu _{t}(dy)}[f(y)-f(x)] \end{aligned} \end{aligned}$$

Further, the decomposition $\nu _t(dr,dx) = \mu _t(dx)dr$ and Eq. (4.1) imply that $(\mu _t)_{t\ge 0}$ is also a solution of this equation (6.1) with initial condition $\mu _0$. As a consequence, we obtain $\mu _t(dx)=\Lambda _t(dx)$ for any $t\ge 0$. This ends the proof.

### Results of section 5.1

#### Proof of Proposition 5.1

Let us first notice that the existence and uniqueness of $\nu \in {\mathcal {C}}([0,T],{\mathcal {M}}_1({\mathcal {X}}))$ as the solution of Eq. (5.4) holds since it corresponds to Eq. (4.1) with a mutation kernel $Q(r,x,dy) = \delta _x(dy)$, which satisfies Assumption 2. Let us now consider a $\beta -$Hölder continuous and bounded test function $\varphi \in {\mathcal {C}}^\beta _b({\mathcal {X}})$ such that $\Vert \varphi \Vert _{{\mathcal {C}}^\beta _b({\mathcal {X}})}\le 1$, then

$$\begin{aligned} \frac{d}{dt}\left\{ \langle \nu ^\varepsilon _t,\varphi \rangle - \langle \nu _t,\varphi \rangle \right\}&= N\iint _{{\mathcal {X}}}\theta (r,x)\nu ^\varepsilon _t(dr,dx)\int _{{\mathbb {R}}^d}\alpha (r,x,x+\varepsilon h)m(r,x,dh)\\ &\quad \times \left[ \varphi (r,x+\varepsilon h) - \varphi (r,x) \right] \\&\quad + N^2\iint _{{\mathcal {X}}}\nu ^\varepsilon _t(dr,dx)\iint _{{\mathcal {X}}}\nu ^\varepsilon _t(dr',dy)\\ &\quad \times \lambda ((r,x),(r',y))\alpha (r,y,x)\left[ \varphi (r,y) - \varphi (r,x) \right] \\&\quad - N^2\iint _{{\mathcal {X}}}\nu _t(dr,dx)\iint _{{\mathcal {X}}}\nu _t(dr',dy)\\ &\quad \times \lambda ((r,x),(r',y))\alpha (r,y,x)\left[ \varphi (r,y) - \varphi (r,x) \right] \\&\le N\varepsilon ^{\beta }\iint _{{\mathcal {X}}}\theta (r,x)\nu ^\varepsilon _t(dr,dx)\\ &\quad \times \int _{{\mathbb {R}}^d}\alpha (r,x,x+\varepsilon h)\Vert h\Vert ^\beta m(r,x,dh) \\&\quad + N^2\iint _{{\mathcal {X}}}\nu ^\varepsilon _t(dr,dx)\iint _{{\mathcal {X}}}\nu ^\varepsilon _t(dr',dy)\\ &\quad \times \lambda ((r,x),(r',y))\alpha (r,y,x)\left[ \varphi (r,y) - \varphi (r,x) \right] \\&\quad - N^2\iint _{{\mathcal {X}}}\nu _t(dr,dx)\iint _{{\mathcal {X}}}\nu ^\varepsilon _t(dr',dy)\\ &\quad \times \lambda ((r,x),(r',y))\alpha (r,y,x)\left[ \varphi (r,y) - \varphi (r,x) \right] \\&\quad + N^2\iint _{{\mathcal {X}}}\nu _t(dr,dx)\iint _{{\mathcal {X}}}\nu ^\varepsilon _t(dr',dy)\\ &\quad \times \lambda ((r,x),(r',y))\alpha (r,y,x)\left[ \varphi (r,y) - \varphi (r,x) \right] \\&\quad - N^2\iint _{{\mathcal {X}}}\nu _t(dr,dx)\iint _{{\mathcal {X}}}\nu _t(dr',dy)\\ &\quad \times \lambda ((r,x),(r',y))\alpha (r,y,x)\left[ \varphi (r,y) - \varphi (r,x) \right] . \end{aligned}$$

Since $\theta$ is bounded, $\varphi \in {\mathcal {C}}^\beta _b({\mathcal {X}})$ and $\lambda \alpha \in {\mathcal {C}}^\beta _b({\mathcal {X}}\times {\mathcal {X}})$, we deduce that

$$\begin{aligned} \frac{d}{dt}\left\{ \langle \nu ^\varepsilon _t,\varphi \rangle - \langle \nu _t,\varphi \rangle \right\} \le C_0\varepsilon ^\beta + C_1 \sup _{\begin{array}{c} \psi \in {\mathcal {C}}^\beta _b({\mathcal {X}}) \\ \Vert \psi \Vert _{{\mathcal {C}}^\beta _b({\mathcal {X}})}\le 1 \end{array}}\{\langle \nu ^\varepsilon _t,\psi \rangle - \langle \nu _t,\psi \rangle \} \end{aligned}$$

where $C_0 = N\Vert \theta \Vert _{\infty }\sup _{r,x}\langle m(r,x,\cdot ),\Vert .\Vert ^\beta \rangle$ ($<+\infty$ thanks to Assumption 3) and $C_1 = C_1(N,\Vert \lambda \alpha \Vert _{{\mathcal {C}}^{\beta }_b({\mathcal {X}}\times {\mathcal {X}})})$. As a consequence,

$$\begin{aligned} \langle \nu ^\varepsilon _t,\varphi \rangle - \langle \nu _t,\varphi \rangle&\le C_0\varepsilon ^\beta T + C_1\int _0^t \sup _{\begin{array}{c} \psi \in {\mathcal {C}}^\beta _b({\mathcal {X}}) \\ \Vert \psi \Vert _{{\mathcal {C}}^\beta _b({\mathcal {X}})}\le 1 \end{array}}\{\langle \nu ^\varepsilon _s,\psi \rangle - \langle \nu _s,\psi \rangle \} ds, \forall t\in [0,T] \end{aligned}$$

where we take the supremum with respect to $\phi$ and apply the Gronwall lemma that gives

$$\begin{aligned} \sup _{\begin{array}{c} \psi \in {\mathcal {C}}^\beta _b({\mathcal {X}}) \\ \Vert \psi \Vert _{{\mathcal {C}}^\beta _b({\mathcal {X}})}\le 1 \end{array}}\{\langle \nu ^\varepsilon _t,\psi \rangle - \langle \nu _t,\psi \rangle \} \le C_0\varepsilon ^\beta Te^{C_1 t}. \end{aligned}$$

Taking the supremum with respect to $t\in [0,T]$ ends the proof. $\square$

#### Proof of Proposition 5.2

Notice first that, using Lebesgue’s dominated convergence theorem, we deduce that Equation (5.4) is still true for any bounded and measurable function $\varphi$. Then the first step of the proof of Proposition 5.2 is to show that the solution to (5.4) has the form $\nu _t(dr,dx) = \sum _{i=1}^nw^i_t(r)\delta _{x^i}(dx)dr$ corresponding to a decomposition similar to the one of its initial condition. To this aim, let us introduce a measurable set $B\subset {\mathbb {R}}^d\setminus \{x^1,\ldots ,x^n\}$, then using Eq. (5.4) with the test function $\varphi (r,x)=1\!\!1_{x\in B}$, we obtain

$$\begin{aligned} \nu _t([0,1]\times B)&= N^2\int _0^t ds\iint _{\mathcal {X}}\nu _s(dr,dx)\\ &\quad \times \iint _{\mathcal {X}}\nu _s(dr',dy)\lambda ((r,x),(r',y))\alpha (r,y,x)\big [ 1\!\!1_{y\in B} - 1\!\!1_{x\in B} \big ] \\&\le N^2\Vert \lambda \alpha \Vert _{\infty }\int _0^t\nu _s([0,1]\times B)ds. \end{aligned}$$

Gronwall’s lemma then implies that $\nu _t([0,1]\times B) = 0$, for any $t\ge 0$. We conclude that $\nu _t(dr,dx) = \sum _{i=1}^n\delta _{x^i}(dx)\nu ^i_t(dr)$ for some non negative measures $\nu ^1_t,\ldots ,\nu ^n_t$. Then, for any $i\in \{1,..,n\}$, using again (5.4) with the test function $\varphi (r,x)=1\!\!1_{x=x^i}1\!\!1_{r\in A}$, for a set $A\subset [0,1]$ negligible with respect to the Lebesgue measure, we obtain $\nu _t(A\times \{x^i\}) = \nu _0(A\times \{x^i\}) = 0$ and we conclude by the Radon-Nikodym Theorem that there exists a measurable function $w^i_t$ such that $\nu ^i(dr)=w^i(r)dr$. This finally implies that $\nu _t(dr,dx) = \sum _{i=1}^nw^i_t(r)\delta _{x^i}(dx)dr$.

In addition, for all $a,b\in [0,1]$, $\int _{a}^b\sum _{i=1}^n w^i_t(r)dr = \int _{{\mathcal {X}}}1\!\!1_{r\in [a,b]}\nu _t(dr,dx)=\int _{{\mathcal {X}}}1\!\!1_{r\in [a,b]}\nu _0(dr,dx)=\int _{a}^b\sum _{i=1}^n w^i_0(r)dr=b-a$, which ensures that $\sum _{i=1}^n w^i_t(r)dr$ is the Lebesgue measure for all $t\ge 0$.

We then deduce from (5.4) that for a fixed $i=1,\ldots ,n$ and any test function in ${\mathcal {C}}({\mathcal {X}})$ of the form $\varphi _i(r,x) = \psi (r)\phi _i(x)$ with $\phi _i(x^i)=1$, $\phi _i(x^j)=0$ for all $j\ne i$, $\psi \in {\mathcal {C}}([0,1])$ and $\phi _i\in {\mathcal {C}}({\mathbb {R}}^d)$, we have

$$\begin{aligned} \frac{d}{dt}\int _0^1w^i_t(r)\psi (r)dr&= \frac{d}{dt} \iint _{\mathcal {X}}\varphi _i(r,x)\nu _t(dr,dx) \\&= N^2\sum _{j,k=1}^n\int _0^1w^j_t(r)dr\int _0^1w^k_t(r')dr'\\ &\quad \times \lambda ((r,x^j),(r',x^k))\alpha (r,x^k,x^j)\big [ \varphi _i(r,x^k) - \varphi _i(r,x^j) \big ] \\&= N^2\sum _{j=1}^n\int _0^1w^j_t(r)\psi (r)dr\int _0^1w^i_t(r')dr'\\ &\quad \times \lambda ((r,x^j),(r',x^i))\alpha (r,x^i,x^j) \\&\quad - N^2\sum _{k=1}^n\int _0^1w^i_t(r)\psi (r)dr\int _0^1w^k_t(r')dr'\\ &\quad \times \lambda ((r,x^i),(r',x^k))\alpha (r,x^k,x^i). \end{aligned}$$

It follows that

6.2 $$\begin{aligned} \frac{d}{dt}\int _0^1w^i_t(r)\psi (r)dr&= N^2\int _0^1\psi (r)\sum _{j=1}^n\bigg \{ w^j_t(r)\alpha (r,x^i,x^j)\nonumber \\ &\quad \quad \times \left( \int _0^1\lambda ((r,x^j),(r',x^i))w^i_t(r')dr' \right) \nonumber \\&\qquad - w^i_t(r)\alpha (r,x^j,x^i)\left( \int _0^1\lambda ((r,x^i),(r',x^j))w^j_t(r') dr' \right) \bigg \}dr \end{aligned}$$

which ends the proof. $\square$

*Proof of Corollaries* 5.3*and* 5.4 Assuming that the metapopulation is homogeneous in the sense of Definition 4.3, we take $\psi \equiv 1$ in Eq. (6.2) here above and obtain

$$\begin{aligned} \frac{d\overline{w}^i_t}{dt}&= N^2\int _0^1\sum _{j=1}^n\bigg \{ w^j_t(r)\alpha (x^i,x^j)\left( \int _0^1\lambda (x^j,x^i)w^i_t(r')dr' \right) \\ &\quad - w^i_t(r)\alpha (x^j,x^i)\left( \int _0^1\lambda (x^i,x^j)w^j_t(r') dr' \right) \bigg \}dr \\&= \overline{w}^i_t\sum _{j=1}^n\underbrace{N^2\left\{ \alpha (x^i,x^j)\lambda (x^j,x^i) - \alpha (x^j,x^i)\lambda (x^i,x^j) \right\} }_{=:\, G(x^i,x^j)}\overline{w}^j_t\, ,\, \forall t\ge 0. \end{aligned}$$

Then denote by $S_t = \sum _{j\ne i^\star }\overline{w}^j_t$, then $\overline{w}^{i^\star }_t + S_t = 1$ and it follows that

$$ \frac{dS_t}{dt} = -\frac{d\overline{w}^{i^\star }_t}{dt} = -\overline{w}^{i^\star }_t\sum _{j=1}^nG(x^{i^\star },x^j)\overline{w}^{j}_t = -(1-S_t)\sum _{j\ne i^{\star }}G(x^{i^\star },x^j)\overline{w}^{j}_t \le 0. $$

Since $S_0 = 1-\overline{w}^{i^\star }_0<1$, we deduce that the function $t\mapsto S_t$ satisfies $S_t<1$ for any $t\ge 0$ and is decreasing. As a consequence, $S_t$ admits a limit when $t\rightarrow +\infty$ and, according to the previous equation, this limit satisfies that

$$\begin{aligned} S_\infty \min _{j\ne i^\star }G(x^{i^\star },x^j) \le \lim _{t\rightarrow +\infty }\sum _{j\ne i^\star }G(x^{i^\star },x^j)\overline{w}^j_{t} = 0, \end{aligned}$$

that is $S_{\infty } = 0$ and finally $\overline{w}^{i^\star }_{\infty } = 1$. In other words, the trait value $x^{i^\star }$ invades the metapopulation. $\square$

### Proofs of section 5.2

**Proof of Theorem** 5.5 We use an approach based on arguments of tightness and uniqueness as in the proof of Theorem 4.1, which is relatively classical, which can be directly adapted from the techniques developed in Ethier and Kurtz (1986) and Fournier and Méléard (2004). However, the time scaling introduces some difficulties that we will detail here.

Let us first give details on the sequence of processes $\{(X^\varepsilon _{\cdot /\varepsilon ^2},\nu ^\varepsilon _{\cdot /\varepsilon ^2}),\varepsilon >0\}$. Recall that $\nu ^\varepsilon \in {\mathcal {C}}([0,T],{\mathcal {M}}_1({\mathbb {R}}^d))$ is characterized by the closed equation (5.10) with the initial condition $\nu _0$. Then setting for all $t\ge 0$, $\xi ^\varepsilon _t:= \nu ^\varepsilon _{t/\varepsilon ^2}$, we obtain for any test function $\varphi \in {\mathcal {C}}^{2}_b({\mathcal {X}})$, the space of bounded and twice-differentiable functions on ${\mathcal {X}}$ with bounded derivatives,

$$\begin{aligned}&\frac{d}{dt}\int _{{\mathbb {R}}^d}\varphi (r,x)\xi ^\varepsilon _t(dr,dx) \\&\quad = \frac{N}{\varepsilon ^2}\iint _{{\mathcal {X}}} \xi ^\varepsilon _t(dr,dx)\theta (r,x) \int _{{\mathbb {R}}^d}\alpha (r,x+\varepsilon h,x)\big [ \varphi (r,x+\varepsilon h) - \varphi (r,x) \big ]m(r,x,dh) \\&\qquad + N^2\iint _{{\mathcal {X}}}\xi ^\varepsilon _t(dr,dx)\iint _{{\mathcal {X}}}\xi ^\varepsilon _t(dr',dy)\lambda ((r,x),(r',y))\alpha (r,y,x)\big [ \varphi (r,y) - \varphi (r,x) \big ] \\&\quad = N\iint _{{\mathcal {X}}} \xi ^\varepsilon _t(dr,dx) \theta (r,x)\\&\qquad \times \int _{{\mathbb {R}}^d}\left( \frac{\alpha (r,x+\varepsilon h,x) - \alpha (r,x,x)}{\varepsilon }\right) \left( \frac{\varphi (r,x+\varepsilon h) - \varphi (r,x)}{\varepsilon } \right) m(r,x,dh) \\&\qquad + \frac{N\alpha (r,x,x)}{\varepsilon ^2}\iint _{{\mathcal {X}}}\xi ^\varepsilon _t(dr,dx) \theta (r,x) \int _{{\mathbb {R}}^d}\big [ \varphi (r,x+\varepsilon h) - \varphi (r,x) \big ]m(r,x,dh) \\&\qquad + N^2\iint _{{\mathcal {X}}}\xi ^\varepsilon _t(dr,dx)\\&\qquad \times \iint _{{\mathcal {X}}}\xi ^\varepsilon _t(dr',dy)\lambda ((r,x),(r',y))\alpha (r,y,x)\big [ \varphi (r,y) - \varphi (r,x) \big ] . \end{aligned}$$

Using the Taylor expansion of order 0 with integral remainder for $\alpha$ and $\varphi$ in the first integral, and of order 1 for $\varphi$ in the second integral, we obtain

$$\begin{aligned} \begin{aligned}&\frac{d}{dt}\int _{{\mathbb {R}}^d}\varphi (r,x)\xi ^\varepsilon _t(dr,dx) \\&\quad = N\iint _{{\mathcal {X}}} \xi ^\varepsilon _t(dr,dx)\theta (r,x)\int _{{\mathbb {R}}^d}\left( \int _0^1h\cdot \nabla _2\alpha (r,x+a\varepsilon h,x) da\right) \\ &\qquad \left( \int _0^1h\cdot \nabla _2\varphi (r,x+a\varepsilon h)da \right) m(r,x,dh) \\&\qquad + \iint _{{\mathcal {X}}} \xi ^\varepsilon _t(dr,dx)\theta (r,x)\int _{{\mathbb {R}}^d}\left( \int _0^1(1-a)h^T\cdot \nabla ^2_2\varphi (r,x+a\varepsilon h)\cdot hda \right) m(r,x,dh) \\&\qquad + N^2\iint _{{\mathcal {X}}}\xi ^\varepsilon _t(dr,dx)\iint _{{\mathcal {X}}}\xi ^\varepsilon _t(dr',dy)\lambda ((r,x),(r',y))\alpha (r,y,x)\big [ \varphi (r,y) - \varphi (r,x) \big ] \end{aligned} \end{aligned}$$

where we used that $\alpha (r,x,x) = 1/N$ (see just above Proposition 3.1) and that the kernel *m* is centered, i.e. $\int _0^1hm(r,x,dh)=0$. And finally, we deduce from the boundedness of the derivatives of $\varphi$ and $\alpha$, and Lebesgue’s dominated convergence theorem, that

6.3 $$\begin{aligned} \begin{aligned}&\frac{d}{dt}\int _{{\mathbb {R}}^d}\varphi (r,x)\xi ^\varepsilon _t(dr,dx) \\&\quad = N\iint _{{\mathcal {X}}} \xi ^\varepsilon _t(dr,dx)\theta (r,x)\int _{{\mathbb {R}}^d}\left( h\cdot \nabla _2\alpha (r,x,x) \right) \left( h\cdot \nabla _2\varphi (r,x)da \right) m(r,x,dh) \\&\qquad ~ + \frac{1}{2}\iint _{{\mathcal {X}}} \xi ^\varepsilon _t(dr,dx)\theta (r,x)\int _{{\mathbb {R}}^d}\left( h^T\cdot \nabla ^2_2\varphi (r,x)\cdot h\right) m(r,x,dh) +R^\varepsilon _t \\&\qquad ~ + N^2\iint _{{\mathcal {X}}}\xi ^\varepsilon _t(dr,dx)\iint _{{\mathcal {X}}}\xi ^\varepsilon _t(dr',dy)\lambda ((r,x),(r',y))\alpha (r,y,x)\big [ \varphi (r,y) - \varphi (r,x) \big ], \end{aligned} \end{aligned}$$

such that $\int _0^T|R^\varepsilon _t|dt$ converges to 0 when $\varepsilon \rightarrow 0$, for any $T>0$.

Further, let us denote $Y^\varepsilon _t:=X^\varepsilon _{\cdot /\varepsilon ^2}$ for all $t\ge 0$. According to (5.11), it is a pure jump inhomogeneous Markov process with the following transitions:

$$ x \xrightarrow []{\text {jumps to}}\left\{ \begin{aligned}&x + \varepsilon h &  \text { at rate } \frac{N}{\varepsilon ^2}\theta (z,x)\alpha (z,x+\varepsilon h,x) m(z,x,dh) \\&y &  \text { at rate } N^2\int _0^1\lambda ((z,x),(r',y))\alpha (z,y,x) \xi ^\varepsilon _t(dr',dy). \end{aligned}\right. $$

In other words, if we set for any test function $f\in {\mathcal {C}}_b({\mathbb {R}}^d)$

$$\begin{aligned} {\mathcal {A}}^\varepsilon f(x,\xi )&= \frac{N}{\varepsilon ^2}\theta (z,x)\int _{{\mathbb {R}}^d}\alpha (z,x+\varepsilon h,x)[f(x+\varepsilon h) - f(x) ]m(z,x,dh) \\&\quad + N^2\iint _{{\mathcal {X}}}\lambda ((z,x),(r',y))\alpha (z,y,x)[f(y) - f(x)]\xi (dr',dy), \end{aligned}$$

then the stochastic process defined by

$$\begin{aligned} M^{\varepsilon ,f}_t = f(Y^\varepsilon _{t}) - f(Y^\varepsilon _0) - \int _0^t{\mathcal {A}}^\varepsilon f(Y^\varepsilon _{s},\xi ^\varepsilon _s)ds \end{aligned}$$

is a square integrable Martingale. In addition, notice that for $f\in {\mathcal {C}}^2_b({\mathcal {X}})$,

$$\begin{aligned} {\mathcal {A}}^\varepsilon f(x,\xi )&= N\theta (z,x)\int _{{\mathbb {R}}^d}\left( \frac{\alpha (z,x+\varepsilon h,x)-\alpha (z,x,x)}{\varepsilon }\right) \\ &\quad \left( \frac{f(x+\varepsilon h) - f(x)}{\varepsilon } \right) m(z,x,dh) \\&\quad + \frac{\theta (z,x)}{\varepsilon ^2}\int _{{\mathbb {R}}^d}[f(x+\varepsilon h) - f(x) ]m(z,x,dh) \\&\quad + N^2\iint _{{\mathcal {X}}}\lambda ((z,x),(r',y))\alpha (z,y,x)[f(y) - f(x)]\xi (dr',dy)\\&= N\theta (z,x)\int _{{\mathbb {R}}^d}\left( \int _0^1h\cdot \nabla _2\alpha (z,x+a\varepsilon h,x)da\right) \\ &\quad \left( \int _0^1h\cdot \nabla f(x+a\varepsilon h)da \right) m(z,x,dh) \\&\quad + \theta (z,x)\int _{{\mathbb {R}}^d}\left( \int _0^1(1-a)h^T\cdot \nabla ^2f(x+a\varepsilon h)\cdot h da \right) m(z,x,dh) \\&\quad + N^2\iint _{{\mathcal {X}}}\lambda ((z,x),(r',y))\alpha (z,y,x)[f(y) - f(x)]\xi (dr',dy) \end{aligned}$$

thanks to the Taylor formula applied on $\alpha$ and the test function *f*, and noting that $\int _{{\mathbb {R}}^d}h m(z,x,dh) = 0$. Then, using arguments similar to those in the proof of Theorem 4.1, we derive the tightness of the sequence of laws $\{ {\mathcal {L}}\big ((Y^\varepsilon _{t},\xi ^\varepsilon _t)_{t\in [0,T]}\big ), \varepsilon >0\}$ in the space ${\mathcal {P}}({\mathbb {D}}([0,T],{\mathbb {R}}^d\times {\mathcal {M}}_1({\mathcal {X}})))$ of probability distributions on the Skorohod space ${\mathbb {D}}([0,T],{\mathcal {M}}_1({\mathcal {X}}))$. The measure space ${\mathcal {M}}_1({\mathcal {X}})$ is endowed with the weak topology. We also derive that subsequential limits ${\mathcal {L}}\big ((X_{t},\xi _t)_{t\in [0,T]}\big )$ exist, by Prokhorov’s Theorem, have the initial condition $(X_0,\xi _0)$ and are such that

$$\begin{aligned} \frac{d}{dt}\int _{{\mathcal {X}}}\varphi (r,x)\xi _t(dr,dx)&= N\iint _{{\mathcal {X}}}\theta (r,x)\left( \Sigma (r,x)\cdot \nabla _2\alpha (r,x,x) \right) \cdot \nabla _2\varphi (r,x)\xi _t(dr,dx) \\&\quad + \frac{1}{2}\iint _{{\mathbb {R}}^d}\theta (r,x)\sum _{ij}\Sigma _{ij}(r,x)\partial _{x_ix_j}\varphi (r,x) \xi _t(dr,dx) \\&\quad + N^2\iint _{{\mathcal {X}}}\xi _t(dr,dx)\iint _{{\mathcal {X}}}\xi _t(dr',dy)\\ &\quad \lambda ((r,x),(r',y))\alpha (r,y,x)\big [\varphi (r,y) - \varphi (r,x) \big ] \end{aligned}$$

for any $\varphi \in {\mathcal {C}}^{0,2}_b({\mathcal {X}})$, and for any $f\in {\mathcal {C}}^2_b({\mathbb {R}}^d)$ the stochastic process

$$\begin{aligned} M^f_t = f(X_t) - f(X_0) - \int _0^t{\mathcal {A}}f(X_s,\xi _s)ds, \forall t\in [0,T] \end{aligned}$$

where

$$\begin{aligned} {\mathcal {A}}f(x,\xi )&= N\theta (z,x)\left( \Sigma (z,x)\cdot \nabla _2\alpha (z,x,x)\right) \cdot \nabla f(x) + \frac{\theta (z,x)}{2}\sum _{ij}\Sigma _{ij}(z,x)\partial _{x_ix_j}f(x) \\&\quad + N^2\iint _{{\mathcal {X}}}\lambda ((z,x),(r',y))\alpha (z,y,x)[f(y) - f(x)]\xi (dr',dy), \end{aligned}$$

is a càd-làg martingale. All that remains is to show that such a subsequential limit is unique, which will allow us to conclude that the initial sequence converges.

Let us first take a look at the equation satisfied by the limit value $\xi$ here above, which also corresponds to Eq. (5.14) in Theorem 5.5 since

$$\begin{aligned} \nabla _2\alpha (r,x,x)&= \frac{-1}{\left( 1 + \sum _{k=1}^{N-1}\left( \frac{c(r,y,x)}{c(r,x,y)} \right) ^k \right) ^2}\sum _{k=1}^{N-1}k\left( \frac{c(r,y,x)}{c(r,x,y)} \right) ^{k-1}\\ &\quad \left. \frac{c(r,x,y)\nabla _2c(r,y,x) - c(r,y,x)\nabla _3c(r,x,y)}{c^2(r,x,y)}\, \right| _{y=x} \\&= -\frac{1}{N^2}\left( \sum _{k=1}^{N-1}k \right) \frac{\nabla _2c(r,x,x) - \nabla _3c(r,x,x)}{c(r,x,x)} \\&= \frac{N-1}{2N} \nabla _2\textrm{Fit}(r,x,x)\, . \end{aligned}$$

Notice that for any test function $(t,r,x)\mapsto \phi _t(r,x)$ of class ${\mathcal {C}}^{1,0,2}$ on $[0,T]\times {\mathcal {X}}$, Eq. (5.14) becomes

$$\begin{aligned} \int _{{\mathcal {X}}}\phi _t(r,x)\xi _t(dr,dx)&= \int _{{\mathcal {X}}}{\phi _0(r,x)}\xi _0(dr,dx) \\ &\quad + \int _0^t ds\iint _{\mathcal {X}}\xi _s(dr,dx)\left\{ \frac{\partial \phi _s(r,\cdot )}{\partial s} + {\mathcal {G}}^{(r)}\phi _s(r,\cdot ) \right\} (x) \\&\quad + \int _0^tds\iint _{{\mathcal {X}}}\xi _s(dr,dx)\iint _{{\mathcal {X}}}\xi _s(dr',dy)\\ &\quad \lambda ((r,x),(r',y))\alpha (r,y,x)\big [ \phi _s(r,y) - \phi _s(r,x) \big ]. \end{aligned}$$

where ${\mathcal {G}}^{(r)}$ is the infinitesimal generator of the strong solution $X^{(r)}$ of the SDE (5.12) without the jump part, with $z=r$, which is unique thanks to Assumption 6. This equation holds as soon as $\frac{\partial \phi _s(r,\cdot )}{\partial s} + {\mathcal {G}}^{(r)}\phi _s(r,\cdot )$ and $\phi _s(r,y)-\phi _s(r,x)$ are bounded according to all variables. By considering in particular the semi-group of $X^{(r)}$ applied to functions of the form $\varphi (r,\cdot )$, defined by

$$\begin{aligned} (t,x)\mapsto P^{(r)}_t\varphi (x) = {\mathbb {E}}_x\left[ \varphi (r,X^{(r)}_t) \right] , \forall t\in [0,T], \forall \varphi \in {\mathcal {C}}_b({\mathcal {X}}), \end{aligned}$$

the function $(s,r,x)\mapsto P^{(r)}_{t-s}\varphi (x)$ is a solution of the system

6.4 $$\begin{aligned} \left\{ \begin{aligned}&\frac{\partial \phi _s(r,\cdot )}{\partial s} + {\mathcal {G}}^{(r)}\phi _s(r,\cdot ) = 0,\,\forall r\in [0,1],\,\forall s< t \\&\phi _t(r,\cdot ) = \varphi (r,\cdot ) \end{aligned} \right. \end{aligned}$$

when $\varphi \in {\mathcal {C}}^{0,2}_b({\mathcal {X}})$ and $t\in (0,T]$. We deduce that $\xi$ satisfies the mild equation

$$\begin{aligned} \int _{{\mathcal {X}}}\varphi (r,x)\xi _t(dr,dx)&= \int _{{\mathcal {X}}}P^{(r)}_t\varphi (x)\xi _0(dr,dx) \\&\quad + \int _0^tds\iint _{{\mathcal {X}}}\xi _s(dr,dx)\iint _{{\mathcal {X}}}\xi _s(dr',dy)\\&\qquad \lambda ((r,x),(r',y))\alpha (r,y,x)\big [ P^{(r)}_{t-s}\varphi (y) - P^{(r)}_{t-s}\varphi (x) \big ]. \end{aligned}$$

for any $\varphi \in {\mathcal {C}}^{0,2}_b({\mathcal {X}})$, that we extend to all $\varphi \in {\mathbb {L}}^\infty ({\mathcal {X}})$ by a regularizing argument, thanks to the Lebesgue’s dominated convergence theorem. Assuming now that there are two limit values $\xi ^1,\xi ^2$, it follows that for any test function $\varphi \in {\mathbb {L}}^{\infty }({\mathcal {X}})$ with $\Vert \varphi \Vert _{\infty }\le 1$ we obtain

$$\begin{aligned}&\iint _{{\mathcal {X}}}\varphi (r,x)(\xi ^1_t-\xi ^2_t)(dr,dx) \\&\quad = \int _0^tds\iint _{{\mathcal {X}}}\xi ^1_s(dr,dx)\iint _{{\mathcal {X}}}\xi ^1_s(dr',dy)\\ &\qquad \times \lambda ((r,x),(r',y))\alpha (r,y,x)\big [ P^{(r)}_{t-s}\varphi (y) - P^{(r)}_{t-s}\varphi (x) \big ] \\&\qquad - \int _0^tds\iint _{{\mathcal {X}}}\xi ^2_s(dr,dx)\iint _{{\mathcal {X}}}\xi ^2_s(dr',dy)\\ &\qquad \times \lambda ((r,x),(r',y))\alpha (r,y,x)\big [ P^{(r)}_{t-s}\varphi (y) - P^{(r)}_{t-s}\varphi (x) \big ] \\&\quad \le C\int _0^t\Vert \xi ^1_s-\xi ^2_s\Vert _{TV}ds \end{aligned}$$

where $\Vert \cdot \Vert _{TV}$ is the total variation norm. Taking the supremum with respect to $\varphi$ and using the Gronwall lemma, we conclude that $\xi ^1=\xi ^2$. The limit process $\xi \in {\mathcal {C}}([0,T],{\mathcal {M}}_1({\mathcal {X}}))$ is then unique.

We are now interested in the limit process *X*. From its description here above we deduce thanks to Lepeltier and Marchal (1976) or Chapter 5.4 in Karatzas and Shreve (2014) that *X* is a càd-làg jump diffusion process described by the SDE (5.12) that is

$$\begin{aligned} dX_t = \frac{N-1}{2}\theta (z,X_t)\Sigma (z,X_t)\cdot \nabla _2\textrm{Fit}(z,X_t,X_t)dt + \sqrt{\theta (z,X_t)}\sigma (z,X_t)\cdot dB_t \end{aligned}$$

(where *B* is a $d-$dimensional Brownian motion), with an additional jump part with kernel

$$\begin{aligned} x \xrightarrow []{\text { jumps to }} y \text { at rate }N^2\int _0^1\lambda ((z,x),(r,y))\alpha (z,y,x)\xi _t(dr,dy). \end{aligned}$$

The strong uniqueness of *X* follows directly from the uniqueness of $\xi$ and Assumption 6 that ensures strong uniqueness for the SDE.
